# TILT TEST TOLERANCE AFTER TWO-WEEKS BED REST IN OLDER ADULTS IS NOT PROTECTED BY DAILY AEROBIC AND RESISTANCE EXERCISE

**DOI:** 10.1093/geroni/igac059.1196

**Published:** 2022-12-20

**Authors:** Andrew Robertson, Richard Hughson, Eric Hedge, Carmelo Mastrandrea, Federico Granados Unger

**Affiliations:** University of Waterloo, Waterloo, Ontario, Canada; Schlegel-UW Research Institute for Aging, Waterloo, Ontario, Canada; Schlegel-UW Research Institute for Aging, Waterloo, Ontario, Canada; Schlegel-UW Research Institute for Aging, Waterloo, Ontario, Canada; Schlegel-UW Research Institute for Aging, Waterloo, Ontario, Canada

## Abstract

Bed rest is associated with increased risk of orthostatic intolerance on return to upright posture and, at least in young adults, regular exercise during bed rest is proposed as protective. 22 older adults (55-65 years, half women) participated in a randomized trial with 14-days of continuous 6-degree head-down bed rest. Half completed 60 min/day of aerobic and high-intensity interval cycling plus resistance exercises (Ex) while the other half were control (Con). A passive 80-degree head-up tilt (HUT) to a maximum of 15-min assessed orthostatic tolerance while cardio- and cerebrovascular responses were monitored to a pre-syncopal level of systolic blood pressure < 90 mmHg. In pre-bed rest baseline, all but 3 women (Ex) completed 15-min HUT. After 14-days bed rest, only 3 men (1 Ex) and 1 woman (Ex) completed 15-min with no differences in tolerance between men and women or Ex and Con. Vasovagal syncope presented in 5 participants (2 M-Con, 1 M-Ex, 1 F-Con, 1 F-Ex), most other non-finishers had progressive reduction in stroke volume without adequate vasoconstriction. Reductions in cerebral blood velocity measured during HUT by Doppler ultrasound revealed strong linear relationships to both end-tidal PCO2 and mean arterial pressure across all participants. These data indicate that neither older men nor older women were protected from orthostatic intolerance by 60-min per day exercise including high-intensity interval training. These data contrast with previous findings in younger adults in controlled trials of bed rest pointing to the need for greater understanding of mechanisms regulating blood pressure and brain blood flow.

